# The unseen risk: a systematic review of the incidence, management, and outcomes of the trigeminocardiac reflex in skull-base surgery

**DOI:** 10.1007/s10143-026-04573-7

**Published:** 2026-11-11

**Authors:** Mohammadmahdi Sabahi, Mohammad Mirahmadi Eraghi, Omid Yousefi, Bhavika Gupta, AmirAli Rastegar Kazerooni, Safwan Alomari, Sara Mostafavi, Serdar Rahmanov, Qais Alrashidi, Seyed Amir Hossein Javadi, Badih Adada, Hamid Borghei-Razavi

**Affiliations:** 1 Department of Neurological Surgery, Pauline Braathen Neurological Center, Cleveland Clinic Florida, Weston, FL USA; 2 Brain and Spinal Cord Injury Research Center, Neuroscience Institute, Tehran University of Medical Sciences, Tehran, Iran; 3 Student Research Committee, School of Medicine, Qeshm International Branch, Islamic Azad University, Qeshm, Iran; 4 Department of Neurosurgery, Shiraz University of Medical Sciences, Shiraz, Iran; 5 Department of Neurosurgery, Allegheny Health Network, Pittsburgh, PA USA; 6 Cleveland Clinic Lerner College of Medicine of Case Western Reserve University School of Medicine, Cleveland, OH USA

**Keywords:** Trigemino-cardiac reflex, Management, Outcome, Systematic review

## Abstract

Purpose: This study synthesizes contemporary evidence on the trigeminocardiac reflex (TCR) during skull-base and adjacent neurosurgical procedures, with emphasis on operative corridors, triggers, hemodynamic phenotypes, management, and the limits of current outcome evidence. Methods: Following PRISMA guidelines, we systematically searched PubMed, Scopus, Web of Science, and Embase through September 15, 2024. Eligible reports described intraoperative TCR or closely related trigeminal autonomic responses during skull-base surgery or adjacent procedures and reported event features, management, or outcomes. Results: Twenty-eight studies including 329 patients met inclusion criteria. Reported TCR events clustered in microvascular decompression for trigeminal neuralgia (n=168; 51.1%), CPA/posterior fossa or anterior skull-base tumor surgery (n=71; 21.6%), transsphenoidal pituitary procedures (n=47; 14.3%), and percutaneous trigeminal procedures (n=35; 10.6%). Immediate cessation of the provoking maneuver was the cornerstone of management, with anticholinergics, vasoactive agents, or temporary pacing required only in selected severe or recurrent cases. No TCR-attributable mortality was reported. However, evidence regarding long-term functional outcomes remains limited, inconsistently reported, and largely dependent on historical vestibular schwannoma and pituitary cohorts. A procedure-specific synthesis identifies high-risk maneuvers, preventive considerations, and escalation options for each operative corridor. Conclusions: TCR should be anticipated during trigeminal manipulation across skull-base surgical corridors and managed initially by immediate cessation or modification of the provoking stimulus, with pharmacologic or resuscitative escalation reserved for persistent or severe events. Although the available evidence supports a procedure-specific approach to prevention and preparedness, heterogeneous definitions, retrospective designs, and incomplete denominators preclude reliable incidence estimates or validated management recommendations.

## Introduction

The trigeminocardiac reflex (TCR) is an autonomic brainstem reflex triggered by stimulation of trigeminal afferents and expressed most commonly as abrupt bradycardia and hypotension, with rare progression to severe bradyarrhythmia or asystole [1, 2]. Although the reflex has been described in craniofacial, ophthalmologic, and skull-base procedures, its incidence and clinical significance remain difficult to interpret because definitions, reporting practices, and denominators vary substantially across studies [1, 3].

For skull-base surgeons and neuroanesthesiologists, the clinically relevant issue is not the general anatomy of the trigeminal nerve, but how reflex events cluster around specific corridors and maneuvers. Manipulation near the trigeminal root entry zone, Meckel’s cave, cavernous sinus, falx, nasal mucosa, or trigeminally innervated dural and vascular territories may provoke reflex hemodynamic instability, yet the magnitude and consequences of these events are inconsistently reported.

This systematic review therefore focuses on the aspects most relevant to perioperative decision-making: operative corridor, provoking maneuver, hemodynamic phenotype, immediate management, and reported postoperative outcomes. Particular attention is given to the methodological limitations of the literature, including publication bias, lack of standardized TCR definitions, and the limited evidence linking intraoperative TCR to long-term functional outcomes. To improve bedside utility, we also synthesize the pathophysiologic basis, modifiable perioperative factors, preventive measures, and a corridor-specific response framework while explicitly grading these recommendations against the limitations of the evidence.

## Materials and methods

This systematic review was conducted to investigate the incidence, management, and outcomes of the TCR during skull-base surgery. The methodology adhered to the Preferred Reporting Items for Systematic Reviews and Meta-Analyses (PRISMA) guidelines.

### Search strategy

A comprehensive search was conducted across PubMed, Scopus, Web of Science, and Embase to identify studies discussing TCR in skull-base surgery and closely related neurosurgical, endoscopic, endovascular, and craniofacial procedures. The search terms included combinations of keywords such as “trigeminocardiac reflex,” “trigemino-cardiac reflex,” “skull-base surgery,” “intraoperative bradycardia,” “asystole,” and “cranial nerve reflexes.” Searches were limited to English-language articles available up to September 15, 2024.

### Study selection

All identified records were imported into reference management software, and duplicates were removed. Titles and abstracts were screened independently by two reviewers based on predefined eligibility criteria. Full-text articles were then assessed for inclusion. Studies were considered eligible if (1) they reported on the occurrence of TCR in the context of skull-base surgery, (2) included data on patient characteristics, surgical approaches, and intraoperative events, and (3) documented outcomes related to TCR management or recovery. Exclusion criteria included studies not related to skull-base surgery, non-human studies, and studies without sufficient data on TCR-related outcomes. Discrepancies in study selection were resolved by consensus or consultation with a third reviewer.

### Data extraction

Data extraction was performed by two independent reviewers using a predefined form. Extracted variables included study design, year, patient demographics, pathology, operative corridor or approach, number of TCR events, denominator availability, operational TCR definition, provoking maneuver, hemodynamic phenotype, management strategy, need for pharmacologic or resuscitative intervention, and reported postoperative outcomes. We specifically recorded whether studies reported functional outcomes such as hearing preservation, tinnitus, cranial nerve deficits, neurological morbidity, or endocrine outcome.

### Definition

No universally accepted gold-standard definition of TCR exists. For this review, we extracted and reported each study’s operational criteria rather than forcing all reports into a single definition. Classical depressor TCR was generally defined as a sudden decrease in heart rate and/or mean arterial blood pressure, most commonly using a > = 20% threshold, although some reports used a 10% threshold, an absolute heart-rate cutoff such as < = 60 bpm, or qualitative descriptions of bradycardia, hypotension, or asystole. We also recorded pressor or hypertensive trigeminal autonomic responses when they were clearly time-locked to trigeminal manipulation. This approach was selected to minimize selection bias while acknowledging that definitional heterogeneity limits direct comparison across studies.

### Risk of bias assessment

Among the included studies, quality of case series and cohorts were assessed using the Methodological Index for Non-Randomized Studies (MINORS) [4], which evaluates both comparative and non-comparative studies. Additionally, the methodological quality of case reports was evaluated using Murad et al.’s quality assessment tool for case reports [5], which covers the domains of selection, ascertainment, causality, and reporting. Two independent reviewers scored each cohort and case-series study based on the 12 MINORS criteria (maximum score 16 for non-comparative and 24 for comparative studies), and independently applied Murad’s tool to the case reports. Discrepancies were resolved through consultation with the senior author, who was the corresponding author in this case.

### Data synthesis

Given clinical and methodological heterogeneity in operative corridor, pathology, anesthetic regimen, monitoring practice, denominator reporting, and TCR definition, we performed a narrative synthesis. Studies were grouped by operative corridor (CPA/posterior fossa, transsphenoidal, anterior/middle fossa or extradural skull base, percutaneous trigeminal procedures, endovascular procedures, and peripheral craniofacial procedures) and by pathology where appropriate. Within each group, we summarized provoking maneuvers, hemodynamic phenotype, management, and reported outcomes.

### Statistical analysis

We reported descriptive statistics at the study level. For studies with explicit denominators, we calculated crude proportions to contextualize incidence signals. We did not pool incidence estimates, calculate between-study variance, or perform meta-analysis because of inconsistent diagnostic thresholds, heterogeneous procedures and populations, small sample sizes, and frequent absence of denominator data. Therefore, all incidence estimates should be interpreted as descriptive signals rather than precise population-level risks.

## Results

### Study selection

**Fig. 1:**
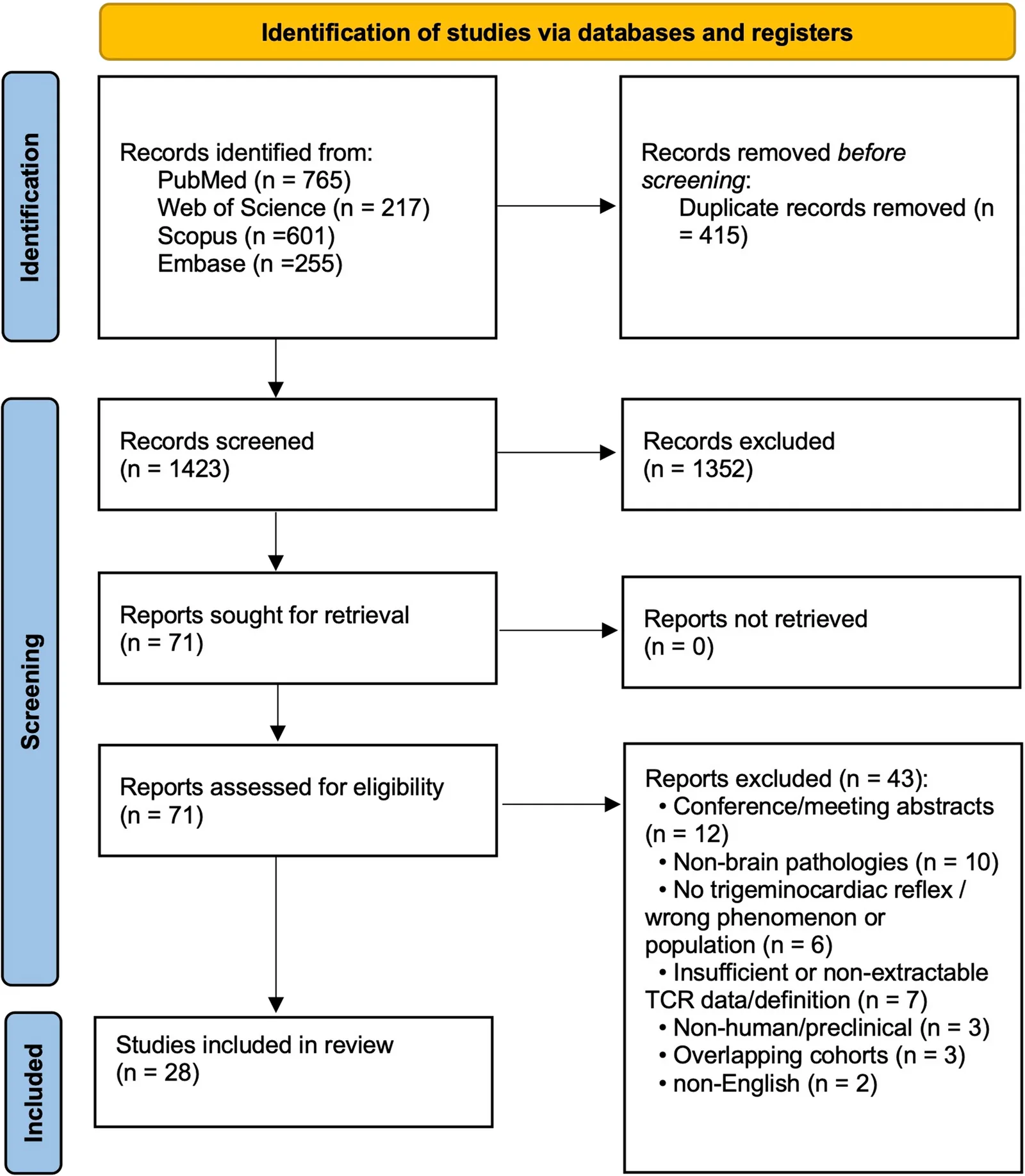
PRISMA flow diagram for study selection

A total of 1,838 articles were retrieved by searching PubMed, Scopus, Web of Science, and Embase through September 15, 2024. After duplicate removal, 1,423 records remained for title/abstract screening. Of the 71 articles taken to full-text review, 43 were excluded for the following reasons: conference/meeting abstracts (*n* = 12), non-brain pathologies (*n* = 10), no TCR/wrong phenomenon or population (*n* = 6), insufficient or non-extractable TCR data/definition (*n* = 7), non-human/preclinical (*n* = 3), overlapping cohorts (*n* = 3), and non-English (*n* = 2). Overall, 28 studies met the inclusion criteria and were included in the systematic review. Figure 1 displays the PRISMA flow diagram depicting the screening process for the papers.

### Study characteristics

As demonstrated in Table 1, the 28 included studies [1, 6–32] describe 329 patients who experienced intraoperative TCR or closely related trigeminal depressor responses during cranial or craniofacial procedures. Among patients with reported age and gender, the weighted mean age was 56.3 years (range 16 to 75 years) and 61% of patients were female. Pathologies were dominated by trigeminal neuralgia (TN) cohorts (203/329; 61.7%), followed by skull-base tumors (59/329; 17.9%, primarily vestibular schwannoma and meningioma) and pituitary adenomas (47/329; 14.3%). The remaining cases comprised vascular lesions (dural arteriovenous fistula, AVM, and aneurysm; 5/329; 1.5%), cluster headache treated with sphenopalatine ganglion (SPG) implantation (3/329; 0.9%), temporomandibular joint dislocation (1/329; 0.3%), and a small number of other supratentorial or craniofacial lesions. When grouped by operative corridor, microvascular decompression (MVD) for TN accounted for just over half of all patients (168/329; 51.1%), followed by CPA/posterior fossa and anterior skull-base tumor craniotomies (71/329; 21.6%). Pituitary/transsphenoidal procedures (microscopic and endoscopic) comprised 47 patients (14.3%), and percutaneous trigeminal procedures (micro-compression and balloon compression of the trigeminal ganglion) accounted for 35 patients (10.6%). Endovascular embolization, SPG implantation, maxillofacial surgery, and other cranial craniotomies together contributed fewer than 3% of the pooled cohort. Eleven of the 28 studies were single-patient case reports, with the remainder being small retrospective case series.

**Table 1:** Study characteristics

Study	Number of patients (M: F)	Age(mean/range)	Underlying pathology	Surgical intervention/approach	Cardiac events	Changes
Brown et al. 1988 [7]	21 (6:15)	62	Trigeminal Neuralgia	Percutaneous micro-compression of the trigeminal ganglion	Hypotension in 12 procedures, Bradycardia in 16 procedures	Mean pre-op HR 76 ± 14 reduced to 55 ± 22 during procedure
Seker et al. 2007 [24]	1 (-:1)	53	Pituitary adenoma	Transnasal-transseptal tumor resection	Sudden asystole	Flatline ECG for 20 s
Schaller et al. 2007 [23]	6 (4:2)	60	Vestibular schwannomas	Suboccipital/retrosigmoid approach	Hypotension and Bradycardia	Drop in MBP and HR more than 20% compared with the baseline
Faraj, et al. 2008 [9]	1 (1:-)	50	Non-functioning pituitary macroadenoma	Two-Step surgery: -transsphenoidal, pterional craniotomy	Bradycardia, asystole, and hypotension	The patient had an HR of 29 and a BP of 44/17 on TCR onset
Acioly et al., 2010 [6]	4 (2:2)	40.5	Non-vestibular schwannoma	Two patients underwent a pre- and retrosigmoid approach. Retrosigmoid approach with a suprameatal extension patient a retro-sigmoid and far lateral retrocondylar approach	Hypotension	A decrease in The MABP of 20% or more is associated with bradycardia lower than 60 beats/min.
Nöthen et al. 2010 [19]	19 (12:7)	51	Pituitary adenoma	Trans-sphenoidal 6 (31.6%), transcranial 1 (5.3%)	Hypotension	A decrease in The MABP of 20% or more is associated with bradycardia lower than 60 beats/min.
Goyal et al. 2012 [10]	2 (2:0)	32.5	Acoustic neuroma - dermoid cyst	Case 1: Retromastoid suboccipital craniotomy Case 2: Midline suboccipital craniotomy	Case 1: Bradycardia and asystole Case 2: Bradycardia	Case 1: transient bradycardia resolved spontaneously, recurred bradycardia proceeded to asystole, Fifteen minutes
Takano et al. 2014 [27]	1 (1:-)	38	Metastatic lesion in the right parietal lobe	Tumor detachment from the falx and resection	Sudden bradycardia/asystole	Cardiac asystole during tumor detachment from the falx cerebri spontaneously resolved. Repeated asystole upon cessation of falcine manipulation
Khatibi et al. 2016 [13]	1 (1:-)	16	Large cerebellar AVM	Endovascular Onyx embolization	Bradycardic and proceeded to asystole	Resuscitation was done due to asystole
Nicholson et al. 2016 [18]	1 (1:-)	59	Dural arteriovenous fistula involving the transverse sinus and torcula	Endovascular Onyx embolization	Sudden asystole	Asystole occurred in the first step of the Onyx injection of the 3-step
Wu et al. 2018 [29]	23 (4:19)	50.12	Trigeminal Neuralgia	Microvascular decompression	hypotension and bradycardia	MBP and HR drop at least 20% with a baseline of 97.2 and 68, respectively
Kim et al.2019 [14]	1 (-:1)	74	Temporomandibular joint dislocation	Open reduction for temporomandibular joint dislocation	Bradycardia	HR drops to 15 beats/min for 30 s
Yazama et al.2020 [31]	1 (-:1)	75	Chordoma-like tumor in the brainstem	Tumor resection through the infratemporal fossa and anterior Petrosal approaches	Sudden asystole	Severe bradycardia and asystole for 10 s
Liu et al. 2020 [16]	70 (26:44)	60.6	Trigeminal Neuralgia	Microvascular decompression	Rise in MBP in 59 cases	Mean rise of 38.8 in BP
Hammad et al. 2020 [12]	3 (NR)	NR	Cluster headache	Sphenopalatine ganglion implant placement	Case 1: hypotension and bradycardia Case 2: 2 episodes of bradycardia Cases3: bradycardia	Case 1: HR of 45 and BP of 88/51 Case 2: HR drops to 38 and later 34 Case 3: HR drops to 41
Recinos et al. 2021 [20]	1 (-:1)	63	Parafalcine meningioma	Tumor detachment from the falx and resection (Two-step surgery in six years)	ST elevation/ Bradycardia	First surgery: sustained ST elevations before dural opening Second surgery (6 years later): profound bradycardia and asystole occurred again
Wang et al. 2021 [28]	2 (2:0)	51.5	Pituitary adenoma	Endoscopic transsphenoidal tumor resection	Bradycardia, hypotension, and asystole	Case 1: asystole and MBP drop, Case 2: irregular rhythm,31 beats/min
Sun et al. 2021 [26]	2 (2:0)	56.5	AVF	Endovascular Onyx embolization	Bradycardia and BP change	Case 1: drop from 113/59 mmHg to 96/49 mmHg and decreased quickly from 61 bmp/min to 43 bmp/min, Case 2: increased blood pressure from 122/73 mmHg to 155/84 mmHg and 68 bmp/min to 46 bmp
Yamada et al. 2021 [30]	1 (1:0)	51	Aneurysmal subarachnoid hemorrhage	Aneurysm clipping through Right frontotemporal (pterional) craniotomy	Bradycardia, asystole	Asystole during opening the dura and during dura manipulation
Zhang et al.2022 [32]	14 (NR)	NR	Trigeminal neuralgia	Percutaneous balloon compression (2% lidocaine 0.5 mL or an equal volume of normal saline was injected into Meckel’s cave)	Bradycardia	Control group (Saline): 8 moderate and 6 severe Bradycardia - Study group (lidocaine): 0 bradycardia
Gupta et al. 2024 [11]	74 (25:49)	65	Trigeminal neuralgia	Microvascular decompression	Classical TCR (*n* = 2): Hypotension. Hypertensive TCR (*n* = 72): Hypertension (BP ≥ 140/90).	Classical TCR: Presented with hypotension. Hypertensive TCR: Mean intraoperative systolic BP during nerve manipulation was 153.1 ± 20.2 mmHg. Specific intraoperative management for the TCR events (e.g., cessation of manipulation, drug administration) was not detailed for this cohort. The occurrence of the hypertensive TCR was a significant predictor of improved surgical outcomes, associated with a lower (better) postoperative Barrow Neurological Institute pain score.
Koerbel et al. (2005) [15]	16 (6:10)	52.12	Vestibular schwannomas (7), sphenoid wing meningiomas (5), petroclival meningiomas (3), intracavernous epidermoid cyst (1)	Retrosigmoid craniectomies (9), retrosigmoid with suprameatal extension (1) frontotemporal craniotomies (6)	Bradycardia (< 60 bpm) + hypotension (MABP drop ≥ 20%) during trigeminal nerve manipulation	Hemodynamic normalization within 20 s after cessation of manipulation; no anticholinergics needed
Schaller et al., 1999 [1]	14 (5:9)	44	- Vestibular schwannomas: 10 (6 Antoni Type A, 4 Type B) - Meningiomas: 4 (2 psammomatous, 1 meningothelial, 1 fibroplastic)	Retrosigmoid approach for CPA tumor resection; intraoperative manipulation near the trigeminal nerve at the brainstem.	Incidence: 11% (14/125) Definition: Bradycardia (HR ≤ 60 bpm) Hypotension (MABP drop ≥ 20%) Asystole/Arrhythmia: 3 cases (transient, resolved post-manipulation)	HR: ↓38% (mean 76 → 47 bpm) during manipulation; returned to baseline. MABP: ↓48% (mean 84 → 44 mmHg) during manipulation; returned to baseline. Treatment: Atropine (0.6–1.2 mg IV) resolved TCR in all cases. Postoperative Complication: 1 patient developed transient sinus arrhythmia (resolved)
Meng et al., 2008 [17]	12 (4:8)	45	Acoustic neuroma: 4 Petroclival meningioma: 3 Sphenoid wing meningioma: 2 Trigeminal neuroma: 1 Trigeminal neuralgia: 1 Traumatic optic neuropathy: 1	- Retrosigmoid approach: 6 - Subtemporal-transzygomatic approach: 2 - Pterionalapproach (with extradural anterior clinoidectomy): 3 - Presigmoid approach: 1	Incidence: 12% (12/100) Definition: Bradycardia (HR drop ≥ 20%) Hypotension (MABP drop ≥ 20%) Severity: HR ↓38% (mean 79 → 49 bpm) MABP ↓33% (mean 93 → 60 mmHg)	Resolution: Spontaneous (8 patients (66%)) Required atropine (4 patients (33%); with a dose of 1.05 ± 0.31 mg) Recurrence: Repeated TCR in 4 cases (2–3 episodes). Postoperative: No sequelae
Schaller et al., 2008 [22]	1 (-:1)	29	Micro-prolactinoma	Transsphenoidal approach (nasal mucosa preparation phase)	Bradycardia (HR ↓20%: 70 → 50 bpm) Hypotension (MABP ↓20%: 130/70 → 100/40 mmHg) ECG Changes: Shortened QT interval	Resolution: Spontaneous normalization after cessation of nasal mucosa manipulation. Atropine administered; no further TCR episodes. Postoperative: Uneventful; no arrhythmias
Chowdhury 2016 [8]	19 (12:7)	51 ± 2.9	Pituitary Adenomas: Hormone-inactive: 10 (53%) Acromegaly: 5 (26%) Cushing’s Disease: 3 (16%) Prolactinoma: 1 (5%)	Transsphenoidal: 18 (95%) Transcranial: 1 (5%)	Simultaneous drop of ≥ 20% in MABP and HR from baseline during tumor manipulation. Incidence: 19 out of 338 patients (6%).	10/19 (52.6%) episodes resolved after anticholinergic administration. 9/19 (47.4%) terminated spontaneously after cessation of stimulation. No asystole or cardiac arrest reported. Hemodynamics normalized promptly in all cases. The proportion of patients with normal pituitary function decreased significantly from 42% preoperatively to 37% postoperatively.
Shibao 2017 [25]	14 (3 : 11)	51 ± 16.63	Meningioma: 12 (85.7%) Schwannoma: 1 (7.1%) Epidermoid Cyst: 1 (7.1%)	Anterior transpetrosal approach	Onset of bradycardia (HR < 60 bpm) + hypotension (≥ 20% reduction in MAP). Frequency: Mean 1.6 ± 1.08 episodes per patient. Asystole occurred in 2 (14.3%) patients.	Cessation of surgical manipulation only: 7 (50%) patients Administration of Atropine: 7 (50%) patients Outcome: Postoperative TN deficit: 4 patients (28.6%) No postoperative hearing disturbances No intraoperative deaths No mortality
Schaller 2007 [21]	4 (NA)	Range: 30–50	Pituitary adenoma	Transsphenoidal surgery via sublabial–transseptal approach	TCR is defined as a ≥ 20% drop in HR and MABP during trigeminal manipulation. Occurred during (1) nasal septum preparation (maxillary branch) and (2) cavernous sinus dissection (branches of the trigeminal nerve).	Maximal intraoperative MABP: 89.9 mmHg (range 81–114) Minimal intraoperative MABP: 45.5 mmHg (range 42–69) Hemodynamic parameters returned to normal within 20 s after cessation of stimulus. Intravenous atropine (0.6 mg) was administered in illustrative cases, after which the TCR was no longer reproducible. No atropine was needed in the general TCR cohort; cessation of stimulus alone was sufficient.

*TCR* trigeminocardiac reflex , *MABP* mean arterial blood pressure based on mmHg, *dAVF* dural arteriovenous fistula, *AVM* arteriovenous malformation, *HR* heart rate, *bpm* based on beats per minute

The evidence base was dominated by case reports and small retrospective series. Therefore, the observed distribution of events should be interpreted as a map of reported TCR contexts rather than as a reliable estimate of true procedure-specific incidence. Severe or dramatic events, such as profound bradycardia or asystole, are likely overrepresented compared with mild transient bradycardia that resolves immediately and may not be published.

### Operational contexts and triggers

Across corridors, TCR episodes were consistently time-locked to maneuvers that stimulate central or proximal trigeminal afferents. In posterior fossa and CPA tumor surgery, events typically occurred during dissection near the trigeminal root entry zone or Meckel’s cave. In percutaneous balloon or micro-compression procedures, TCR was most often elicited during Meckel’s cave cannulation and balloon inflation. In transsphenoidal pituitary surgery, episodes clustered around nasal mucosal preparation, sphenoid and sellar work, and cavernous sinus dissection. Anterior and middle skull-base exposures (e.g., anterior transpetrosal approach, extradural anterior clinoidectomy) produced TCR when drilling or dissecting near the trigeminal nerve or ganglion, whereas supratentorial falcine/parafalcine tumors triggered reflex events during tumor detachment from the falx cerebri. Endovascular series documented bradycardia, hypotension, or asystole during Onyx injection into dural arterial feeders, implicating dural branches of the trigeminal system. Craniofacial and peripheral procedures—SPG implantation and TMJ open reduction—demonstrated that stimulation of more distal branches (V2/V3) can also activate the reflex, although these contexts remain less commonly reported.

### Hemodynamic phenotype

Definitions were inconsistent across the included studies. Many reports used a > = 20% decrease in HR and/or MABP, some used an absolute HR threshold such as < = 60 bpm, and case reports often relied on qualitative descriptions of bradycardia, hypotension, or asystole. This variability introduces measurement noise and limits cross-study comparability. The classical phenotype was abrupt bradycardia with hypotension, most often during CPA/posterior fossa tumor surgery, transsphenoidal procedures, and percutaneous trigeminal procedures. However, pressor or mixed responses were also reported, particularly in MVD and internal neurolysis cohorts, suggesting that the broader trigeminal autonomic spectrum may include both depressor and hypertensive phenotypes. Rare asystolic or high-grade bradyarrhythmic events were described in transsphenoidal surgery, falcine detachment, endovascular Onyx embolization, and aneurysm clipping.

### Incidence signals by procedure

Because denominators and operational definitions differed substantially, incidence estimates should be viewed as corridor-specific signals rather than pooled risks. In CPA/posterior fossa tumor surgery, series using a depressor definition generally reported TCR in the range of approximately 8–12%. In transsphenoidal pituitary surgery, reported rates were approximately 6–10%, with events often occurring during nasal preparation, sphenoid or sellar work, or manipulation of invasive adenomas. Percutaneous trigeminal compression procedures frequently elicited bradycardia during Meckel’s cave cannulation or balloon inflation, and one randomized trial suggested that trigeminal ganglion block with lidocaine can markedly reduce bradycardia. MVD cohorts were heterogeneous because some focused on classic depressor responses whereas others emphasized hypertensive variants. Endovascular and falcine cases confirm that trigeminally innervated dural or vascular territories can trigger TCR even outside traditional open skull-base corridors.

### Management and intraoperative course

Across the pooled literature, immediate cessation of the provoking maneuver was the cornerstone of management and typically led to normalization of HR and blood pressure within seconds. In many series, particularly CPA tumor and transsphenoidal cohorts, the majority of episodes resolved without pharmacologic intervention once the surgeon paused dissection or reduced traction. When hemodynamic instability persisted or recurred, anticholinergic agents—most commonly atropine 0.6–1.2 mg IV—were effective in restoring HR and MABP and, in several reports, rendered the reflex non-reproducible when manipulation resumed. A minority of cases with asystole or high-grade bradyarrhythmia required brief cardiopulmonary resuscitation or temporary pacing (including transcutaneous pacing in one illustrative aneurysm-clipping case). Endovascular and SPG-implantation reports emphasize pre-briefing with anesthesia, stepwise escalation of stimulation (e.g., cautious Onyx injection, graded SPG lead advancement), and ready access to vasoactive support. Some series also explore preventive strategies, including local anesthetic instillation (ganglion block in Meckel’s cave, intra-arterial lidocaine before embolization), although these approaches remain supported largely by small non-randomized cohorts and a single randomized trial.

The resulting patient-care synthesis is summarized in Table 2, which links each procedure or corridor to the most reproducible trigger, practical preventive considerations, and an immediate technical response. These entries are intended as cognitive aids for the operative team and not as estimates of comparative treatment efficacy.

**Table 2:** Procedure-specific triggers, preventive considerations, and intraoperative response pearls for the trigeminocardiac reflex

Procedure/corridor	High-risk stimulus	Preparation/prevention	Immediate response and technical pearl
CPA/posterior fossa tumor surgery; MVD or internal neurolysis	Dissection at the trigeminal root entry zone or Meckel’s cave; nerve combing; abrupt or sustained traction	Pre-brief before trigeminal contact; continuous ECG and blood-pressure surveillance; gentle, intermittent manipulation; distinguish depressor from isolated pressor responses	Stop and release the stimulus. Resume only after full recovery and with less force or traction. Treat persistent bradycardia/hypotension; atropine is not appropriate for an isolated pressor phenotype
Percutaneous trigeminal microcompression or balloon compression	Foramen ovale cannulation, Meckel’s cave entry, and balloon inflation	Announce cannulation/inflation before each stimulus; monitor closely during both steps; trigeminal ganglion block is supported by one randomized study but is not yet generalizable	Pause cannulation or deflate the balloon; allow hemodynamics to normalize; if reattempted, use graded inflation and treat persistent/recurrent depressor responses
Microscopic or endoscopic transsphenoidal surgery	Nasal preparation or packing; sphenoid/sellar work; manipulation of invasive adenoma or cavernous sinus	Warn anesthesia before nasal and cavernous-sinus stimulation; use graded manipulation; verify oxygenation, ventilation, and adequate anesthetic depth	Stop the nasal or sellar maneuver and remove traction/packing when feasible. Escalate to anticholinergic/vasoactive support or resuscitation for persistent severe events
Anterior/middle skull-base and transpetrosal approaches	Drilling or dissection near trigeminal branches, ganglion, Meckel’s cave, or adjacent dura	Identify high-risk phases in the team brief; drill and dissect incrementally; avoid prolonged traction	Pause drilling/dissection, release traction, and resume stepwise only after recovery; change the operative plane or maneuver if events recur
Falcine or parasagittal surgery	Tumor detachment from the posterior falx or traction on trigeminally innervated dura	Forewarn anesthesia before falcine detachment; use incremental traction and dissection	Release the falx/dural stimulus; stabilize hemodynamics; consider an alternative dissection plane if the response is reproducible
Endovascular embolization	Onyx or other embolic injection in trigeminally innervated dural feeders or near the cavernous sinus	Pre-brief before injection; use slow, staged delivery with continuous monitoring; intra-arterial lidocaine has only limited procedure-specific evidence	Stop the injection immediately; provide hemodynamic support; modify injection rate or position before any rechallenge; reserve local-anesthetic strategies for selected cases.
SPG implantation and maxillofacial procedures	Lead advancement near V2/SPG; maxillary or mandibular stimulation; TMJ traction	Use graded lead advancement or tissue manipulation and announce each high-risk step	Withdraw the lead or release traction; reattempt only after normalization and with reduced stimulation; treat persistent depressor responses.
Other cranial procedures, including aneurysm surgery	Reproducible dural, cavernous-sinus, or trigeminal stimulation; rare severe bradyarrhythmia/asystole	When prior severe or reproducible events are known, agree on rescue drugs and pacing/resuscitation readiness before re-exposure	Remove the stimulus first. For refractory asystole or high-grade bradyarrhythmia, initiate advanced resuscitation and temporary pacing when indicated.

*ECG *electrocardiography,* MVD *microvascular decompression,* SPG *sphenopalatine ganglion

### Outcomes

Across all 28 studies, intraoperative TCR events were usually transient, and no TCR-attributable mortality was reported. Most patients experienced prompt intraoperative recovery after cessation of the provoking maneuver, with or without anticholinergic or vasoactive therapy. However, postoperative functional outcomes were inconsistently reported and rarely analyzed with comparator groups. Existing data suggest possible associations between intraoperative TCR-associated hypotension and worse hearing preservation or tinnitus after vestibular schwannoma surgery, and some pituitary series reported modest postoperative endocrine shifts. Nevertheless, the available evidence does not support strong causal conclusions because event severity, duration, recurrence, anesthetic covariates, and postoperative functional endpoints were not systematically captured across studies.

### Risk of bias assessment

The methodological quality of the included studies was generally moderate, as assessed using the MINORS tool (Table 3), while case reports were appraised using Murad’s tool (Table 4). The most important limitations were retrospective design, small sample size, absence of prospective sample-size calculations, incomplete denominator reporting, and inconsistent outcome ascertainment. These limitations reduce confidence in procedure-specific incidence estimates and make functional-outcome analysis largely exploratory.

**Table 3:** Quality assessment of cohorts and case series using the methodological index for non-randomized studies (MINORS)

First author, year	A clearly stated aim	Inclusion of consecutive patients	Prospective collection of data	Endpoints appropriate to the aim of the study	Unbiased assessment of the study endpoint	Follow-up period appropriate to the aim of the study	Loss to follow-up less than 5%	Prospective calculation of the study size	Adequate control group	Contemporary groups	Baseline equivalence of groups	Adequate statistical analyses	Total score
Schaller, 1999	2	2	2	2	2	1	2	2	N/A	N/A	N/A	N/A	15
Koerbel, 2005	1	2	2	2	1	1	2	2	N/A	N/A	N/A	N/A	13
Schaller, 2007	1	0	0	0	0	0	1	0	N/A	N/A	N/A	N/A	2
Schaller, 2008	1	2	1	1	2	1	2	2	N/A	N/A	N/A	N/A	12
Meng Q, 2008	2	2	2	2	2	0	2	2	N/A	N/A	N/A	N/A	14
Brown, 1988	2	1	0	2	0	2	1	0	N/A	N/A	N/A	N/A	8
Acioly, 2010	2	2	0	2	0	2	2	0	N/A	N/A	N/A	N/A	10
Nöthen, 2010	2	2	0	2	0	2	2	0	N/A	N/A	N/A	N/A	10
Hammad, 2020	2	2	0	2	0	1	2	0	N/A	N/A	N/A	N/A	9
Goyal, 2012	2	0	0	2	0	2	2	0	N/A	N/A	N/A	N/A	8
Wang, 2021	2	0	0	2	0	2	2	0	N/A	N/A	N/A	N/A	8
Sun, 2021	2	0	0	2	0	2	2	0	N/A	N/A	N/A	N/A	8
Zhang, 2022	2	2	2	2	2	0	2	2	2	2	2	2	22
Gupta, 2024	2	2	2	2	2	0	2	2	2	2	2	2	22
Shibao, 2017	2	2	2	2	2	0	2	2	2	2	2	2	22
Chowdhury, 2015	2	2	2	2	2	0	2	2	2	2	2	2	22
Wu, 2018	2	2	0	2	0	2	2	0	2	2	2	2	18
Liu, 2020	2	2	0	2	0	2	2	0	2	2	2	2	18

**Table 4:** Quality appraisal of the included case report using Murad’s quality assessment tool [1], summarizing item-level judgments across all methodological domains

Domains	Leading explanatory questions	Seker (2009)	Faraj (2008)	Takano (2014)	Khatibi (2017)	Nicholson (2018)	Kim (2019)	Yazama (2020)	Recinos (2021)	Yamada (2021)	Schaller (2008)
Selection	1. Does the patient(s) represent(s) the whole experience of the investigator (centre) or is the selection method unclear to the extent that other patients with similar presentation may not have been reported?	1. Yes	1. Yes	1. Yes	1. Yes	1. Yes	1. Yes	1. Yes	1. Yes	1. Yes	1. Yes
Ascertainment	2. Was the exposure adequately ascertained? 3. Was the outcome adequately ascertained?	2. Yes 3. Yes	2. Yes 3. Yes	2. Yes 3. Yes	2. Yes 3. Yes	2. Yes 3. Yes	2. Yes 3. Yes	2. Yes 3. Yes	2. Yes 3. Yes	2. Yes 3. Yes	2. Yes 3. Yes
Causality	4. Were other alternative causes that may explain the observation ruled out? 5. Was there a challenge/rechallenge phenomenon? 6. Was there a dose–response effect? 7. Was follow-up long enough for outcomes to occur?	4. Yes 5. NA 6. NA 7.Yes		4. Unclear 5.Yes 6.Unclear 7.Yes	4. Unclear 5.Yes 6. NA 7.Yes	4. Yes 5.Yes 6.Unclear 7.Yes	4. Yes 5.Yes 6.Unclear 7.Yes	4. Unclear 5.Yes 6.Unclear 7.Yes	4. Yes 5.Yes 6.Unclear 7.Yes	4. Yes 5.Yes 6.Unclear 7.Yes	4. Yes 5.Yes 6.Unclear 7.Yes
Reporting	8. Is the case(s) described with sufficient details to allow other investigators to replicate the research or to allow practitioners make inferences related to their own practice?	Yes	Yes	Yes	Yes	Yes	Yes	Yes	Yes	Yes	Yes

## Discussion

This systematic review shows that reported TCR events are primarily maneuver-linked rather than diagnosis-specific. Events cluster around surgical corridors that bring instruments, traction, drilling, balloon inflation, or embolic material into contact with central or proximal trigeminal afferents, including the root entry zone, Meckel’s cave, cavernous sinus, falx, nasal mucosa, and trigeminally innervated dural or vascular territories. The main contribution of this review is the systematic aggregation of these reported events across classic microscopic skull-base corridors as well as modern endoscopic, percutaneous, and endovascular approaches.

The hypertensive or pressor variant of trigeminal autonomic response deserves separate consideration. Unlike classic depressor TCR, which produces bradycardia and hypotension, the hypertensive variant may present with transient increases in blood pressure during trigeminal manipulation and has been reported in MVD cohorts. In the available MVD literature, these pressor responses may correlate with postoperative pain outcomes, but they should not be interpreted as equivalent to classic vagal depressor TCR. Excessive hypertension may carry hemorrhagic risk, and prophylactic atropine is not appropriate for this sympathetic phenotype [11].

### Pathophysiologic basis

The afferent limb begins with mechanical, chemical, or thermal stimulation of sensory endings in any trigeminal division. Impulses traverse the Gasserian ganglion to the trigeminal sensory nucleus; short internuncial fibers in the brainstem reticular formation then engage vagal motor nuclei. The efferent response combines parasympathetic cardioinhibition with reduced sympathetic vasomotor tone in the classic depressor phenotype [1, 33–35]. This dual output explains why an anticholinergic may correct bradycardia while hypotension persists and requires separate vasoactive support.

The anatomic site and intensity of stimulation help explain the observed corridor dependence. Central or proximal manipulation at the root entry zone, Meckel’s cave, cavernous sinus, or trigeminally innervated dura can provoke abrupt depressor responses; peripheral V1-V3 stimulation and mixed trigeminal-autonomic responses may be more variable. Abrupt or sustained traction appears more provocative than gentle intermittent manipulation. Hypoxemia, hypercarbia, and insufficient anesthetic depth are described susceptibility factors in TCR or closely related oculocardiac and peripheral trigeminal reflex literature, although available data do not permit reliable individual risk prediction [1, 35–37].

A key interpretive problem is publication bias. The included literature relies heavily on case reports and retrospective case series, which preferentially capture unusual, severe, or dramatic events such as profound bradycardia, asystole, or resuscitation. Mild transient bradycardia that resolves immediately after pausing dissection is less likely to be reported. Consequently, the published literature probably overestimates the severity of TCR while still underestimating its true frequency in routine practice.

Definitional heterogeneity is an additional barrier to synthesis. Some studies required simultaneous decreases in HR and MABP, others used either variable alone, and thresholds ranged from 10% to 20% or qualitative descriptions. Some reports also included pressor responses within the broader trigeminal autonomic spectrum. This heterogeneity explains why formal meta-analysis was not appropriate and why our results should be interpreted as a descriptive synthesis of reported events rather than a pooled incidence estimate.

Risk mitigation begins with anticipating high-risk maneuvers and maintaining direct communication between the surgeon and anesthesia team. Sudden or sustained traction appears more likely to trigger the reflex than gentle, graded manipulation [1]. Hypoxemia, hypercarbia, and light anesthesia may further increase susceptibility, particularly in related oculocardiac or peripheral trigeminal reflexes [36, 37]. Immediate cessation or reduction of the provoking stimulus remains the most consistent intervention, and multiple reports describe rapid normalization of hemodynamics after the maneuver is paused [33].

### Procedure-specific prevention and preparedness

Before a high-risk maneuver, the surgeon should identify the anticipated trigger and explicitly notify the anesthesia team; the team should document baseline HR and blood pressure, verify oxygenation, ventilation, and adequate anesthetic depth, and ensure immediate access to anticholinergic and vasoactive therapy. During exposure, traction, drilling, balloon inflation, lead advancement, or embolic injection should be incremental whenever technically feasible. Routine prophylactic anticholinergic treatment is not supported by the available evidence and may be inappropriate for an isolated pressor response.

Local-anesthetic prevention is procedure-specific rather than universal. A single randomized study supports trigeminal ganglion block during percutaneous balloon compression, whereas intra-arterial lidocaine during endovascular embolization is supported primarily by small reports [26, 32]. These findings justify selective consideration in recurrent or predictable TCR but not routine extrapolation to all skull-base procedures. Table 2 summarizes the procedure-specific triggers and preventive pearls supported by the included literature.

Pharmacologic escalation should be tailored to the phenotype and severity of the event. Anticholinergics such as atropine may be useful for persistent or recurrent bradycardic depressor responses, but atropine alone may not fully prevent TCR because the reflex can combine vagal cardioinhibition with suppression of adrenergic vasoconstriction [34]. Severe events with asystole or high-grade bradyarrhythmia may require brief resuscitative support or temporary pacing. Conversely, episodes that resolve after modifying the surgical maneuver emphasize that correction of the trigger is often more important than medication alone [38].

### Pragmatic intraoperative response pathway

#### Step 1

Announce suspected TCR and immediately stop, release, or reverse the provoking maneuver.

#### Step 2

Confirm hemodynamic recovery while checking oxygenation, ventilation, and anesthetic depth and excluding competing causes such as hemorrhage, venous air embolism, medication effect, or primary arrhythmia.

#### Step 3

If HR and blood pressure normalize promptly, resume only after explicit surgeon-anesthesiologist agreement, using a slower, less forceful, or alternative maneuver with close surveillance for recurrence.

#### Step 4

For persistent or recurrent depressor responses, treat the observed phenotype: use an anticholinergic for clinically significant bradycardia and vasoactive support for hypotension according to anesthetic judgment and local protocols; manage an isolated pressor response separately.

#### Step 5

For asystole or high-grade bradyarrhythmia, initiate advanced resuscitation and temporary pacing when indicated. Repeated severe events despite technical modification should prompt abandonment or postponement of the provoking maneuver.

This sequence is a pragmatic synthesis of recurring actions in the included literature, not a prospectively validated treatment algorithm. Its primary purpose is to shorten recognition-to-stimulus-release time and to structure escalation for the uncommon event that does not resolve immediately.

The relationship between TCR and functional outcome remains underdefined. The strongest historical signal comes from vestibular schwannoma literature, where intraoperative TCR-associated hypotension has been linked to worse hearing preservation and higher postoperative ipsilateral tinnitus [39, 40]. Hearing preservation data have suggested markedly lower rates of preserved auditory function in patients with intraoperative TCR compared with those without it [40, 41]. However, these findings have not been substantially expanded by contemporary prospective datasets, and most included studies did not systematically capture functional endpoints, comparator groups, duration of hemodynamic instability, or recurrence of events.

Accordingly, the functional-outcome implications of TCR should be framed as a persistent evidence gap rather than a definitive conclusion. Future studies should prospectively record the definition used, baseline HR and MABP, anesthetic depth, CO2 status, provoking maneuver, event duration, recurrence, management, and postoperative functional outcomes. Such standardized reporting would allow corridor-specific risk estimation and clarify whether brief TCR episodes are merely transient intraoperative events or clinically meaningful contributors to auditory, cranial nerve, neurological, or endocrine outcomes.

### Limitations

This review has several important limitations. First, the available evidence is dominated by case reports and retrospective case series, creating substantial publication bias. Severe or unusual TCR events, including profound bradycardia, asystole, or cardiac arrest, are more likely to be published than mild transient bradycardia that resolves after pausing the stimulus. Therefore, the published literature may overestimate the apparent severity of TCR.

Second, there is no universal gold-standard definition for TCR. Included studies variably used decreases in HR, decreases in MABP, combined HR/MABP thresholds, absolute HR cutoffs, or qualitative descriptions of bradyarrhythmia. Thresholds also differed, commonly ranging from 10% to 20%. This lack of standardization introduces measurement heterogeneity and prevents reliable pooled meta-analysis.

Third, many studies lacked denominators and key perioperative covariates, including baseline hemodynamics, anesthetic depth, CO2 status, local anesthetic infiltration, anticholinergic prophylaxis, vasoactive support, and duration or recurrence of TCR episodes. These omissions limit procedure-specific incidence estimates and make it difficult to distinguish true TCR from alternative causes of bradycardia or hypotension.

Finally, functional outcomes were inconsistently reported. Most studies did not include comparator groups without TCR, did not quantify the severity or duration of the reflex event, and did not prospectively assess postoperative hearing, tinnitus, cranial nerve function, neurological morbidity, or endocrine outcomes. Consequently, conclusions regarding functional outcome effects should be considered hypothesis-generating.

The procedure-specific preventive recommendations and response pathway are also limited by the same low-certainty evidence. They synthesize repeatedly reported actions and selected procedure-specific studies but have not been prospectively compared with alternative protocols; therefore, they should not be interpreted as formal guidelines or as proof that any one strategy improves long-term outcomes.

## Conclusion

TCR is an important intraoperative phenomenon reported across microscopic, endoscopic, percutaneous, and endovascular approaches. Although usually reversible after cessation or modification of the provoking maneuver, selected episodes require anticholinergic or vasoactive therapy, resuscitation, or temporary pacing. In addition to aggregating reported events by operative corridor and trigger, this review translates those observations into a procedure-specific prevention table and a pragmatic stepwise response pathway for surgeon-anesthesiologist teams.

The current literature remains limited by publication bias, inconsistent diagnostic thresholds, retrospective design, and frequent absence of denominators. Accordingly, the proposed pathway is a clinical aid rather than a validated guideline, and evidence linking TCR to long-term functional outcomes remains limited and largely historical. Future prospective studies should use standardized hemodynamic definitions, record the trigger, event severity, duration, recurrence, and management, and correlate TCR with postoperative functional outcomes.

## Data Availability

The authors confirm that the data supporting the findings of this study are available within the article.

## Declarations

### AI usage

AI-based tools were used to assist with language and editorial editing. All content was verified by the authors, who take full responsibility for the work.
